# Billing I-AIM: a novel framework for ultrasound billing

**DOI:** 10.1186/s13089-020-0157-0

**Published:** 2020-02-28

**Authors:** Daralee Hughes, Michelle M. Corrado, Irene Mynatt, Michael Prats, Nelson A. Royall, Creagh Boulger, David P. Bahner

**Affiliations:** 1 Department of Emergency Medicine, The Ohio State University College of Medicine, 750 Prior Hall, 376 W 10th Ave, Columbus, OH 43210 USA; 2 Children’s Hospital Colorado, The University of Colorado, Denver, CO USA; 3 Department of Surgery, The University of Oklahoma College of Medicine, Tulsa, OK USA

**Keywords:** Point-of-care ultrasound, Ultrasonography, Ultrasound billing, Ultrasound reimbursement, Emergency service, Bedside ultrasound

## Abstract

**Background:**

Point-of-care ultrasound (POCUS) has an ever-growing footprint in medicine. With this growth POCUS billing and reimbursement has become an area gaining quite a bit of attention as a means of funding and sustaining quality and education programs. Standardization across providers is needed to improve the financial viability of POCUS.

**Results:**

We created an institutional collaborative which developed a framework to identify critical POCUS billing and reimbursement checkpoints. The framework, Billing I-AIM, provides a feasible structure to enhance provider-based reimbursement and perform quality improvement efforts across variable POCUS environments.

**Conclusions:**

POCUS billing using the Billing I-AIM technique allows administrative oversight, quality assurance, and educational functions as well. A discussion of the framework and respective application is provided.

## Background

Point-of-care ultrasound (POCUS) utilization in medicine is rapidly increasing in academic and community settings [1]. Numerous studies demonstrate that physician-performed POCUS improves patient care through increased diagnostic accuracy, decreased time to diagnosis and intervention, decreased procedural complications, decreased length of stay, and decreased patient care costs [1–10]. Although utilization of POCUS is increasing, standard procedures for ultrasound documentation and billing procedures have not been codified with standardized terminology in the published medical literature.

The American College of Emergency Physicians (ACEP), as well as organizations from other specialties, have defined the scope of practice for POCUS [10, 11]. These guidelines from 2016 establish four clinical categories for POCUS: resuscitative, diagnostic, symptom or sign-based procedure guidance, and therapeutic and monitoring [3]. Across all clinical categories, POCUS applications are accepted to be a separate billable procedure. To this point, unique codes exist for POCUS within Current Procedural Terminology (CPT) and International Classification of Disease Procedures Coding System (ICD-PCS). POCUS billing requires additional unique documentation and variable preservation of imaging based upon the clinical category and payer source, which differs from comprehensive radiology ultrasound exams. In POCUS, images and interpretations are completed by the same provider at the bedside, whereas comprehensive reports are completed by an ultrasonographer and then interpreted by a radiologist. Published information on billing and reimbursement for POCUS is limited, and many providers report a high rate of reimbursement denials related to this complex billing process unfamiliar to the average provider [1, 5].

As providers are increasingly expected to learn the billing and reimbursement process, there is a need to streamline the billing for reimbursement. Given these challenges, The Ohio State University Wexner Medical Center created an institutional collaborative aimed at evaluating and enhancing the billing and reimbursement process for POCUS. Given the success of teaching mnemonics for acquisition of ultrasound, we proposed similar mnemonics would be beneficial for teaching the billing of POCUS [12]. The purpose of this article is to present concepts developed through the collaborative which facilitate reliable billing for POCUS. We aim to focus on the emergency department (ED) given our institutional experience, but these concepts apply to other hospital-based specialties as well [10].

## Methods: the concept

Previously the concept of using a structured mnemonic to teach and perform POCUS has been published and is widely used. This concept, known as I-AIM, is the basis of the nomenclature in this paper used as a way to understand the requirements for POCUS billing and reimbursement [12]. It explains the components involved in the process of performing POCUS, documentation requirements, coding specifics, reasons for revenue loss, and the ways in which ultrasound scans can be classified based on the presence or absence of images and documentation.

**Fig. 1:**
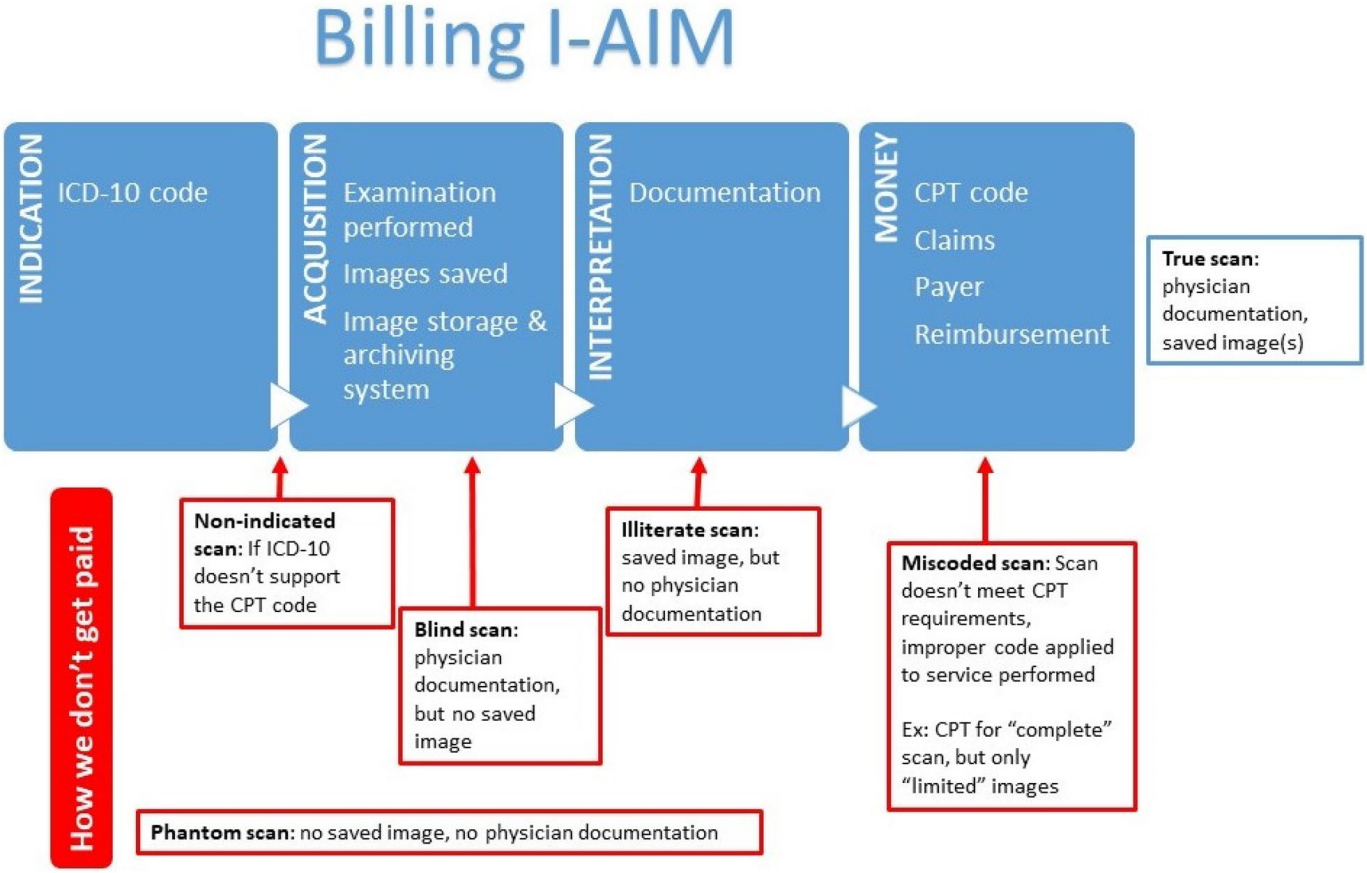
Billing I-AIM is a simplified framework to enhance provider-based reimbursement and identify common errors

The Billing I-AIM concept is (1) Indication, (2) Acquisition, (3) Interpretation, and (4) Money, and includes the components necessary for each of those steps to achieve revenue generation from performed POCUS (Fig. 1). A description of the Billing I-AIM concept is provided in Fig. 1 along with common barriers to reimbursement and nomenclature stratifying ultrasound exams on the presence of documentation and saved images. A discussion of this nomenclature is followed by more detailed information on the components of Billing I-AIM with examples to illustrate concepts.

## Results

### Ultrasound scan nomenclature

POCUS reimbursement relies upon Medicare standards for diagnostic imaging, which require documentation of the exam performed and images or video depicting the exam. A written interpretation of the indication and description of visualized structures and abnormalities, in addition to stored images within a database are recommended by ACEP in order to fulfill Medicare and other payer requirements for ultrasound examinations [13]. The omission of either documentation or stored images potentially leads to lost revenue.

**Fig. 2:**
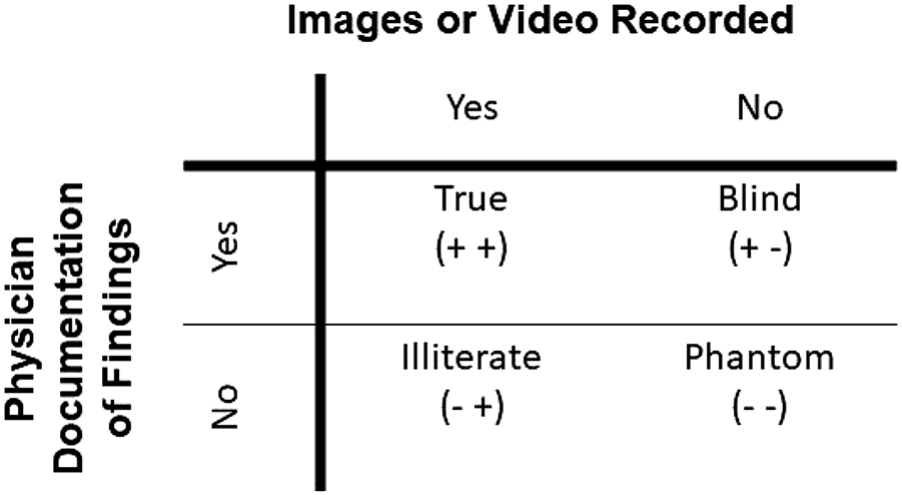
For correct billing, both saved images and documentation of the scanning procedure are necessary

Given Medicare and ACEP criteria for POCUS examination reimbursement, the following nomenclature was developed to discriminate between properly and improperly completed examinations: phantom, blind, illiterate or true scans (Fig. 2).

Phantom scans are those with no saved images and no provider documentation. From a coding, billing, and medicolegal perspective, these scans never occurred. There is no way to bill for the services provided, and no opportunity for quality assurance.

Blind scans are those with no saved images, but with provider documentation of the scan and integration into the medical decision-making. Without images there can be no quality assurance or review by other providers. These scans are not able to be billed.

Illiterate scans are those with saved images, but without provider documentation. These scans have images that are available for quality assurance and patient care, but documentation is missing. Unlike blind scans, illiterate scans can be reviewed, rectified, and documented at a later time by the performing provider. Blind scans are unable to be retrospectively converted to true scans.

True scan is the final classification. These exams include an appropriate indication, saved images and provider documentation in the medical record. True scans can be coded and billed with the expectation of potential revenue generation.

There are additional permutations in the above-mentioned scans that impact the ability to generate revenue. If the patient’s symptoms or diagnosis do not support the ultrasound examination performed, then the scan is not indicated and should not be coded or billed. Miscoded ultrasound exams can also limit revenue. This type of examination does not satisfy the requirements for billing or reimbursement based on the Current Procedural Terminology (CPT) code submitted, which is generally outside of the physician’s ability to control. For example, if an ultrasound examination with images supporting only a limited CPT code is submitted under the complete CPT code, it may not be reimbursed. Details regarding limited versus complete scans and further information on CPT codes are discussed in subsequent sections.

#### Billing for POCUS

There are specific reporting and documentation requirements for POCUS. The requirements are (1) test indication, (2) written report, and (3) interpretation [13]. The ultrasound exam must be medically necessary and the signs, symptoms or diagnoses prompting the ultrasound must be recorded in the EMR. There must be a written report signed by the performing physician for each procedure performed [13]. The ultrasound examination should be immediately interpreted and communicated to other providers via the patient’s medical record. The documentation should describe the structures evaluated, the presence or absence of relevant anatomy or pathology, and the interpretation of findings [3, 13]. Incomplete or improper documentation prevents coding, billing, and physician compensation for patient care provided [5].

#### Indication

The indication for any ultrasound examination performed by the hospital-based physician is the complaint, exam finding, or other test result that demonstrates the medical necessity, which must be properly documented [13]. The protocol-driven use of ultrasound or a “screening examination” (such as for AAA in a patient with risk factors but without abnormal vital signs, classic symptoms or physical exam findings) may not be deemed medically necessary and may be denied payment as a non-indicated procedure performed [13].

Medical necessity is currently determined by the International Classification of Diseases (ICD), currently in the 10th edition. The ICD-10 is a code set published by the World Health Organization and is a cataloging tool developed for comparison of morbidity and mortality data [13]. Each code defines “the medical situation and conveys the necessity for subsequent medical services performed” [14]. The ICD-10 code is used by payers to determine why a procedure was performed. This differs from the CPT code which explains to the payer what procedure or service was performed [13]. The CPT codes are discussed further in the “Money” section.

If the patient’s symptoms, test results, or diagnosis do not support the medical necessity of the performed procedure, or if the ICD-10 code is not covered by the payer, then the examination may be denied payment.

#### Acquisition

The acquisition of ultrasound images by the performing provider should be within the scope of practice as defined by their governing body. In order to perform an ultrasound at an institution, the provider should also be credentialed in this skill. Images and videos should clearly identify the relevant anatomy and any abnormal findings, if present. These ultrasound examinations should be linked to the patient’s medical record. A unique accession number is created to link the ultrasound examination to the medical record. Saved images or recorded videos are then transmitted to an image archival system or database and permanently linked to the patient’s medical record. Indefinite retention of images performed is preferred, but different areas of practice may have different requirements and this should be explored at the specific institution [15].

Ultrasound hardware with wireless connectivity, image archival systems, and integration with EMR allows images acquired to be accessible to other providers as well as coding specialists and payers, if requested [3]. This process is similar to how most radiology images and reports are linked to the patient’s EMR. The use of an ultrasound management system allows administrative oversight, quality assurance, and educational functions as well [3].

The current guidelines specify that at least one image should be permanently retained; however, regulations do not specify the number of images required for reimbursement or the storage system utilized [13]. Improper image acquisition may result in reimbursement delay or denial.

For ultrasound-guided procedures, including vascular access, images must be saved before, during or after the procedure; real-time imaging is recommended but not required [16, 17]. A procedure note should document that ultrasound was used to obtain real-time visualization as well as a dedicated report for the ultrasound images (discussed in more detail below). The ultrasound examination and the procedure should both be billed. Procedures can also be ultrasound-assisted, in which ultrasound is utilized prior to the procedure but not during the procedure.

#### Interpretation

The ultrasound examination should ideally be immediately interpreted and entered into the patient’s medical record. The documentation should describe the structures evaluated, the presence or absence of relevant anatomy or pathology, and the interpretation of findings [3, 13]. Incomplete or improper documentation prevents coding, billing, and provider compensation for patient care provided [5].

The scope of the study performed should be documented and include whether the examination was (1) limited versus complete, (2) a repeat examination by the same or subsequent physician with documentation on why it was repeated, and (3) if there is reduced level of service or any other relevant information regarding the scope of the study [13]. This additional information on the scope of the study performed is vital to proper coding and directly impacts the ability for reimbursement. Interpretation of the ultrasound examination should be completed prior to billing.

**Fig. 3:**
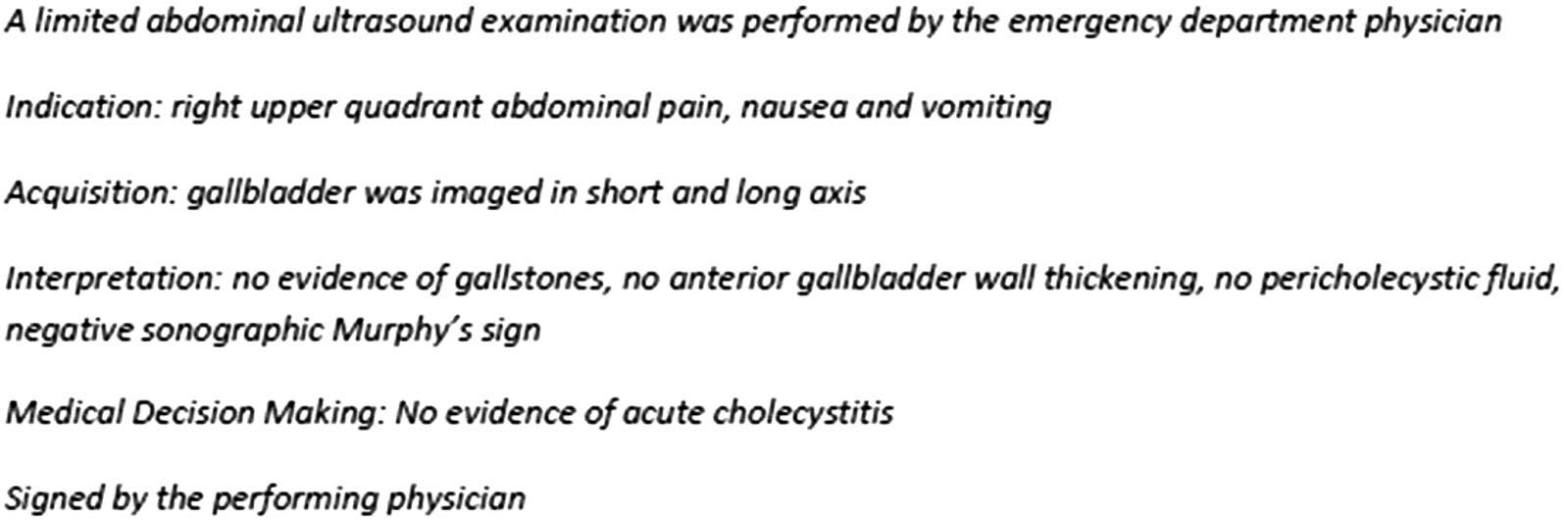
Example of proper documentation following the I-AIM method

**Fig. 4:**
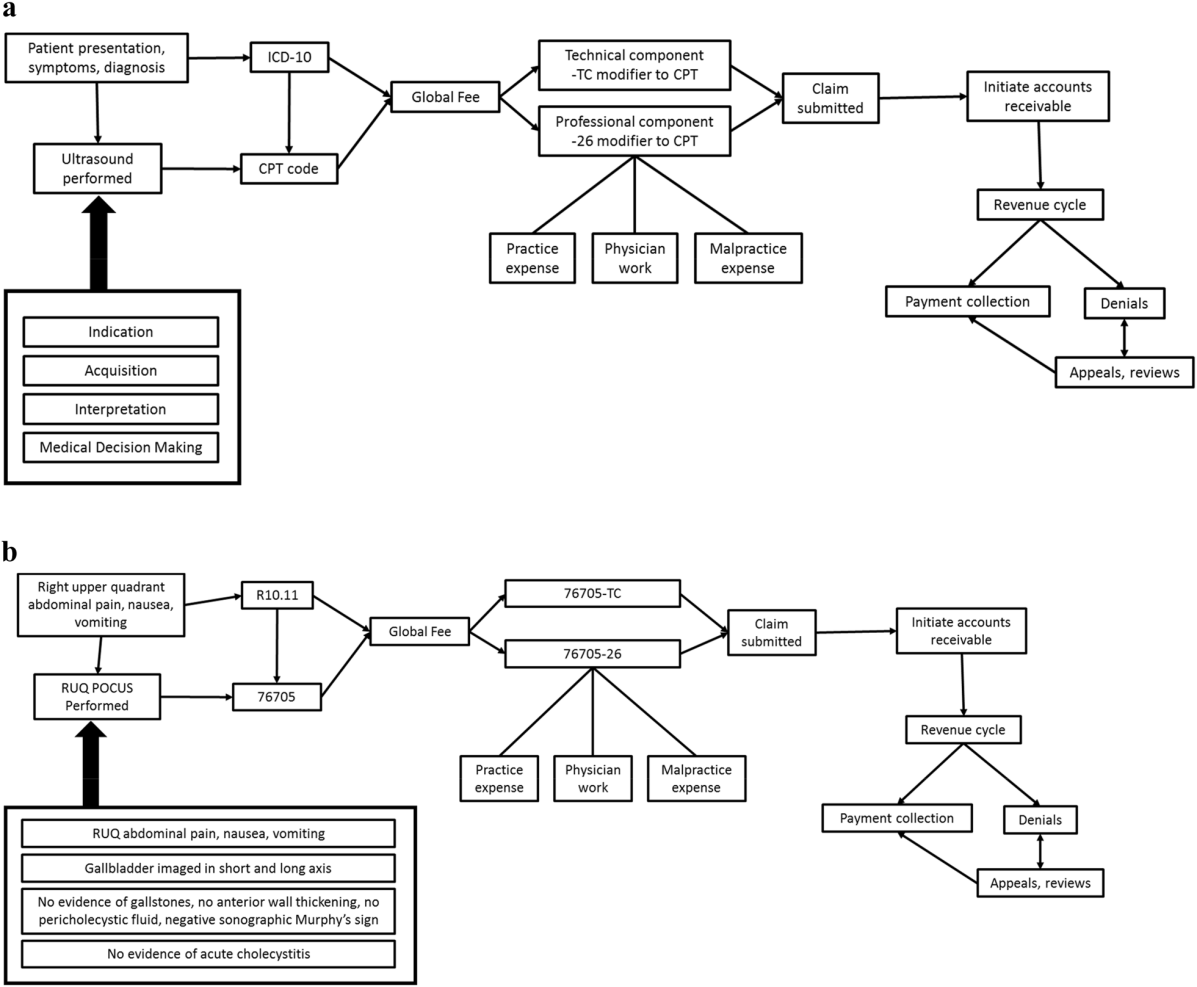
Process flowchart illustrating the Billing I-AIM method in point-of-care ultrasound billing and coding

An example of proper documentation, modified from the previously published I-AIM method [12], and billing process is shown in Figs. 3 and 4.

#### Money

The Centers for Medicare and Medicaid Services (CMS) administers the Medicare and Medicaid programs which currently command the largest market share for healthcare payments [18]. Private payers tend to follow the CMS systems for reimbursement and payment, based on units of work performed (fee-for-service reimbursement system) [18].

CPT codes are a system of descriptive terms to identify and report services that are performed [13]. This standardized code set is a communication tool between providers and payers to relay information about the medical care provided, and is used as a claims-processing tool by payers. Through an established formula, CPT codes attempt to quantify input costs of provider services and help payers determine the amount of reimbursement for services provided by being linked to relative value units (RVUs) to enable prediction of reimbursement [18]. Although the codes are standardized, the amount of reimbursement from the payer varies based on payer and local contracts [18, 19]. Multiple ultrasound CPT codes may be applied to the same patient during a single encounter if medically necessary [13]. See Table 1 for commonly used ultrasound CPT codes in the ED.

**Table 1:** Common current procedural terminology (CPT) codes associated with point-of-care ultrasound

CPT code	Ultrasound study
93308	FAST (focused assessment of sonography in trauma): scan for hemopericardium and hemoperitoneum, may include lung ultrasound^a^
76705	For pneumothorax
76604	
76815	Pregnant transabdominal (TA)
76817	Pregnant transvaginal (TV)
76775	Retroperitoneal: aorta, renal
93308	Cardiac
76604	Thoracic (chest only)
76705	Biliary
76857	Bladder
93971	DVT—unilateral
93970	DVT—bilateral
Soft tissue ultrasound	
76536	Head/neck
76882	Axilla
76604	Chest wall
76641	Breast
76604	Upper back
76705	Lower back
76705	Abdominal wall
76857	Pelvic wall
76882	Extremity
76512	Ocular
76999	Miscellaneous ultrasound
Ultrasound-guided procedure codes^b^	
76930	Ultrasound-guided pericardiocentesis
76937	Ultrasound-guided vascular access placement
32555	Ultrasound-guided thoracentesis
49083	Ultrasound-guided paracentesis
76942	Miscellaneous ultrasound-guided procedure without catheter
76942	Ultrasound-guided abscess drainage
76942	Ultrasound-guided peritonsillar abscess drainage
76942	Ultrasound-guided lumbar puncture
76942	Ultrasound-guided suprapubic aspiration
76942	Ultrasound-guided fb removal
20604	Ultrasound-guided joint aspiration: small joint or bursa—finger, toe
20606	Ultrasound-guided joint aspiration: intermediate joint or bursa—elbow, wrist
20611	Ultrasound-guided joint aspiration: major joint or bursa—shoulder, hip, knee

^a^FAST: not a specific CPT code, FAST is a compilation of separate studies to evaluate areas potentially injured in trauma. Can bill for the specific components performed

^b^Can add the CPT code for the procedural component as well

The revenue cycle, the full discussion of which is beyond the scope of this article, requires that the examination is completed, the report is signed, and the chart is properly coded (ICD-10 and CPT). Then a claim can be submitted initiating an accounts receivable (AR) that ultimately results in revenue collection [18].

##### Limited versus complete

Ultrasound examinations are designated as either limited or complete. A complete examination attempts to visualize and evaluate all anatomic structures within a region and therefore is not routinely performed in POCUS. In contrast, a limited examination is focused on specific anatomic structures or a diagnostic question [19]. For example, a complete abdominal exam (CPT 76700) will evaluate and document the liver, gallbladder, common bile duct, pancreas, spleen, kidneys, upper abdominal aorta, and inferior vena cava, tissue, vascular flow, and pathology. A limited abdominal exam (CPT 76705) could visualize and evaluate the right upper quadrant for signs of acute cholecystitis. If a limited and complete examination of the same anatomic region is performed on a patient during the same encounter, the limited portion is subsumed by the complete examination code [13].

##### Modifiers

A CPT modifier is a two-digit code reported at the end of the five-digit CPT code to further classify the service provided [13]. Multiple modifiers can be reported for a patient if appropriate [13]. Table 2 lists a summary of common CPT modifiers applied for POCUS.

**Table 2:** Common CPT modifier codes, to be added after the five-digit CPT code (see Table 1)

CPT modifier	Description of modifier	Example
26	Professional component of global fee	Physician component of global fee for professional services including interpretation of diagnostic tests with separate signed report
52	Partially reduced service provided	Transvaginal ultrasound in known pregnant patient (76817, no limited code exists), but POCUS does not include all anatomic structures in the region required for billing
59	Distinct procedural service—report procedures that are distinct but have the same CPT code^a^	Multiple soft tissue areas on different extremities examined for abscess vs cellulitis (76882)
76	Repeat procedure by the same physician^b^	Trauma patient requiring a repeat FAST examination by an emergency physician for a change in clinical stability such as hypotension
77	Repeat procedure by another physician^c^	Trauma patient requiring a repeat FAST examination by the trauma surgeon for a change in clinical stability such as hypotension

^a^*CPT* current procedural terminology

^b^Same physician—providers who are under the same group Medicare provider number on the same date of service or patient encounter

^c^Another physician—provider with a different group Medicare provider number on the same date of service or patient encounter

The most commonly applied CPT modifier in POCUS separates the billable expense into a professional and technical component (TC). Emergency physicians cannot bill the combined, professional plus technical, CPT for ultrasound performed in ED unless they directly own the ultrasound equipment [18–20]. In these settings, the physician reports using the -26 professional modifier and the facility reports using the -TC technical component modifier.

##### CPT codes

ICD-10 codes applied to the patient encounter are used by payer sources to determine the medical necessity of reported CPT codes. National and Local Coverage Determinations (LCDs) are lists of pre-approved ICD-10 codes that support using a particular CPT code [13, 18]. If an ICD-10 is reported for an encounter which is listed on the LCD for a CPT code, then the probability of reimbursement is increased. The combination of ICD-10 codes on a particular LCD will vary based on regional Medicare Administrative Contractor (MAC) who developed the LCD for a state or region.

Submitted CPT codes for a particular patient encounter are compared with the ICD-10 diagnosis codes in a process termed Edits. Edits are policy-driven computer programs or manual reviews to check information on payment claim forms to determine coded services which have a high probability of being incorrect or medically unnecessary [13]. The charge is reimbursed through an appeal demonstrating medical necessity; termed a “diagnosis” or “payment” edit [13]. Other types of edits include procedure-to-procedure edits, frequency-to-time edits, and site of service edits. Procedure-to-procedure edits occur when the billed CPT code is compared to a second CPT code to ensure that the reporting of a group of procedures with related codes is given the most appropriate comprehensive code [13]. This occurs if the same physician attempts to bill a limited and complete exam of the same anatomic region during the same visit; the limited exam would be denied as it is subsumed by the complete exam which is the more appropriate and most comprehensive code. Frequency-to-time edits apply when multiple exams of the same anatomic region are performed during the same patient encounter and will be denied unless medical necessity for the repeated exam is documented. These subsequent exams would also require the modifiers as discussed above [13]. The classic example of this would be the blunt abdominal trauma patient who has a negative initial focused assessment with sonography in trauma (FAST) examination, but becomes hemodynamically unstable necessitating a repeat FAST examination to evaluate for intra-abdominal free fluid. Site of service edits are also performed in which the CPT code is compared to the site where the service is performed and if the global CPT code, without the -26 professional component modifier, is billed by a hospital-based emergency physician (EP), it would be rejected [13].

There are additional considerations for CPT codes submitted for standard POCUS examinations such as the FAST exam, soft tissue/musculoskeletal, pelvic ultrasound, and ultrasound-guided procedures. The FAST exam is not a single CPT code but actually two separately billed exams—limited abdomen (CPT 76705) and limited echocardiography (CPT 93308). If not all views of the FAST are performed, only the CPT code that accurately describes the anatomic regions evaluated should be used. Soft tissue and musculoskeletal ultrasound examinations are coded based on the location of the body examined and all are limited examinations, so the reduced service modifier (− 52) is not required [13] Pelvic ultrasound also has some special considerations in regard to CPT codes selected. The initial determination is whether the patient is known, prior to performance of the ultrasound, to be pregnant or not regardless of the final ultrasonographic findings. In general, pelvic ultrasound performed in the ED will be on a “known” pregnant patient to evaluate for intrauterine pregnancy and/or fetal cardiac activity. These CPT codes are 76815 for transabdominal pelvic ultrasound in a pregnant patient and 76817 for transvaginal pelvic ultrasound in a pregnant patient. If both transabdominal and transvaginal examinations are medically necessary, both can be performed, coded and billed. This may be necessary if, in an early first trimester pregnancy, the provider performed transabdominal ultrasound cannot rule-in intrauterine pregnancy and then the same provider performs a transvaginal ultrasound to rule-in intrauterine pregnancy (76817-26,-52). If the provider cannot rule-in intrauterine pregnancy via transabdominal or transvaginal ultrasound, a comprehensive radiology performed transvaginal ultrasound can be ordered to determine if an intrauterine or ectopic pregnancy is present. The provider can bill for both ultrasounds and radiology can bill for the comprehensive transvaginal ultrasound 76817-77 (CPT modifier -77 for repeat examination by second physician with a different group Medicare provider number) [13]. It is inappropriate to routinely perform a transabdominal and then a transvaginal ultrasound on all pregnant patients as each ultrasound performed must be medically necessary.

Ultrasound-guided procedures are another area of special consideration with regard to coding and billing. Diagnostic POCUS and related ultrasound-guided CPT codes can be reported only if the procedural code does not subsume the diagnostic code. For example, if a patient undergoes an ultrasound-guided pericardiocentesis after identifying a pericardial effusion with tamponade with POCUS, the physician should report a limited echocardiography (93308-26), ultrasound-guided pericardiocentesis (76930-26), and pericardiocentesis (33010) [13].

## Discussion

POCUS has rapidly expanded as a standard component of emergency medicine practices. While emergency medicine programs have readily adopted POCUS programs and increasingly adopted quality improvement processes for image review, the development of fiscally sustainable models has been difficult due to the myriad of variables affecting image storage, documentation, coding, denials, and reimbursement. Many POCUS programs within emergency medicine practices currently are either operating without a formal billing process or lack a feasible reimbursement pattern [21, 22].

A lack of understanding regarding the intricacies of billing for POCUS can be an impediment to its use. A program that is able to show evidence of income is more readily supported by administrators concerned with return on investments. Conversely, without any reimbursement from POCUS, it is harder to justify the benefits to those who are not using this valuable modality clinically. It is apparent that POCUS can offer advantage to patients and health systems alike when decreasing costs for the same level of care. Studies have shown improved patient safety when using POCUS for procedures or eliminating the need for a procedure, increased patient satisfaction, improved department resource utilization, reduction in more costly or invasive procedures, and improved clinical decision-making [3, 13]. Unfortunately, many of these benefits will not be realized if there is no initial investment in POCUS programs. A well-organized and successful billing program can be an asset to both developing and established programs.

The Billing I-AIM model creates an understandable outline of the steps that need to take place for reimbursement. At each step there are potential pitfalls and barriers to success. The indication for each POCUS should be clear, including what focused questions are being asked. It is valuable to understand that what constitutes an appropriate indication will be determined based on the diagnosis in the form of ICD-10 codes. Documentation of the indication, and specifically the medical necessity, is especially important for repeat examinations that may necessitate further CPT modifiers. With regard to acquisition, it is necessary to save images in a permanent database. Ideally this will be linked to patients’ medical record. Phantom scans without saved images or documentation are unacceptable and will result in no reimbursement. Having no images saved leads to a blind scan which is also not billable.

The interpretation of the POCUS exam should be well documented. Poor compliance with timely documentation of exams can be a disservice to the patient and other providers caring for them, as well as impeding the billing process. This deserves special attention, because it is a common barrier encountered that can significantly impact billing patterns. An exam without documentation is considered illiterate and non-billable. Reports that are not completed in a timely fashion may not allow coders sufficient documentation to support medical necessity of the exam or include the exam in the billable patient encounter [5]. Certain conditions may improve compliance. Web-based workflow and archival systems with a user-friendly interface can be valuable. It is additionally useful if offering remote access so providers can complete documentation from outside of the work place. Wirelessly transferring images to the system is efficient and advantageous compared to manually exporting and uploading ultrasound exams. Ongoing education and productivity reports distributed to faculty and trainees can increase compliance with workflow requirements. Reminder emails from administrators or ultrasound faculty can also improve completion of charting. Quality assurance is also an important component in ensuring the accuracy of interpretation. This can also be means of providing feedback for future improved quality of acquisition and interpretation. Efforts to improve compliance with documentation of POCUS exams can lead to rectification of these illiterate exams to true billable examinations.

Reimbursement for POCUS can increase the E&M code as well, and thus reimbursement for the ED encounter, by increasing the complexity of the medical decision-making [1]. Reimbursement rates affect the amount of income generated which is dependent on the payer mix of the region in which the ED is located and is also determined by the payers’ concept of accepted professional medical standards, even though these may not always be based on the most current literature [1].

Although the nomenclature described in this article represents an initial attempt to differentiate causes for inadequately documented POCUS scans, there are several limitations. First, use of the nomenclature has not been externally validated to produce a significant difference in identifying quality improvement for POCUS scans within emergency ultrasound programs. A larger and prospective application of the nomenclature within multiple emergency ultrasound programs is required prior to its application for the purposes of quality improvement.

## Conclusions

The Billing I-AIM model serves as both a mnemonic and a checklist for the process of billing a POCUS examination. By breaking down a potentially intimidating process into its core components of indication, acquisition, interpretation, and money, practitioners of POCUS can easily remember and understand the steps necessary for successful reimbursement. The proposed nomenclature allows differentiation of POCUS examinations performed for the purposes of quality improvement and improved reimbursement. Future studies can examine how incorporating this model into a POCUS billing system may increase revenue from POCUS examinations. As successful billing can solidify the longevity of a POCUS program, our hope is that improvement on this front will facilitate further use of this valuable modality.

## Data Availability

Not applicable. Data sharing is not applicable to this article as no datasets were generated or analyzed during the current study.

## Abbreviations

AAA: Abdominal aortic aneurysm
ACEP: American College of Emergency Physicians
CMS: Centers for Medicare and Medicaid Services
CPT: Current Procedural Terminology
E&M: Evaluation and management
EMR: Electronic medical record
IAIM: Indication, Acquisition, Interpretation, Money
ICD-PCS: International Classification of Disease Procedures Coding System
LCD: Local coverage determinations
POCUS: Point-of-care ultrasound
TC: Technical component
TVUS: Transvaginal ultrasound
US: Ultrasound
WHO: World Health Organization
